# Identification of functional lncRNAs in atrial fibrillation based on RNA sequencing

**DOI:** 10.1186/s12872-023-03573-5

**Published:** 2023-11-06

**Authors:** Yangyang You, Wei Wang, Wenshu Zhu, Jian Xu

**Affiliations:** 1 Department of Cardiology, The First Affiliated Hospital of USTC, Division of Life Sciences and Medicine, University of Science and Technology of China, Hefei, Anhui 230001 China; 2 Department of Cardiology, Bengbu First People’s Hospital, Bengbu, Anhui 233000 China

**Keywords:** Long noncoding RNA, Atrial fibrillation, RNA sequencing, *AC009509.2*, Biomarker, Prediction

## Abstract

**Background:**

Atrial fibrillation (AF) is one of the most common arrhythmia contributing to serious conditions such as stroke and heart failure. Recent studies demonstrated that long noncoding RNAs (lncRNAs) were related to cardiovascular disease. However, the molecular mechanisms of AF are not fully clear. This study intended to discover lncRNAs that are differentially expressed in AF compared with controls and evaluate the potential functions of these lncRNAs.

**Methods:**

Ninety-seven patients (49 patients with AF and 48 patients without AF) were included in this study. Among these patients, leucocyte suspensions of 3 AF patients and 3 controls were sent for RNA-seq analysis to select differentially expressed lncRNA and mRNA. Different lncRNA expressions were validated in another samples (46 AF patients and 45 controls). Gene ontology (GO) enrichment analysis was conducted to annotate the function of selected mRNAs. Alternative splicing (AS) analysis was performed and a lncRNA-mRNA network was also constructed. The receiver operating characteristics (ROC) curve was used to evaluate diagnostic values. Logistic regression analysis was utilized to assess the risk or protective factor of AF.

**Results:**

A total of 223 mRNAs and 105 lncRNAs were detected in AF patients compared with controls. Total 4 lncRNAs (*LINC01781*, *AC009509.2*, *AL662844.3*, *AL662844.4*) associated with AF were picked out for validation in another samples by quantitative real-time PCR (qRT-PCR), detecting that upregulated *AC009509.2* and downregulated *LINC01781* in AF patients. Multivariate logistic regression analysis illustrated that left atrial diameter (OR 1.201; 95% CI 1.093-1.320; *P*=0.000) and AC009509.2 (OR 1.732; 95% CI 1.092-2.747; *P*=0.020) were related to AF respectively. ROC curve showed that *AC009509.2*, *LINC01781* and left atrial diameter (LAD) were predictors of AF. For *LINC01781*, the area under the curve (AUC) was 0.654 (95% CI 0.541-0.767, *P*=0.0113). For *AC009509.2*, the AUC was 0.710 (95% CI 0.599-0.822, *P*=0.0005). Bioinformatic methods (GO enrichment, AS analysis and lncRNA-mRNA network construction) were performed to reveal the role of lncRNAs.

**Conclusions:**

This study discussed differentially expressed lncRNA and their potential interaction with mRNA in AF. LncRNA *AC009509.2* could be a new potential biomarker for AF prediction.

**Supplementary Information:**

The online version contains supplementary material available at 10.1186/s12872-023-03573-5.

## Background

Atrial fibrillation (AF) is an irregular and rapid cardiac arrhythmia, which can lead to serious conditions such as stroke and heart failure [1, 2]. A chaotic and diffuse pattern of electrical activity occurs in the atria when AF happens [3]. The molecular mechanisms contributing to atrial remodeling in structural and electrophysiological aspects are complex and have not been completely discovered.

Long noncoding RNAs (lncRNAs) are RNAs longer than 200 nucleotides and lack coding capacity [4]. Emerging research has shown that lncRNAs execute their functions in a variety of biological processes mainly at transcription, post-transcription and epigenetic modification levels [5]. To date, many lines of evidence suggested that lncRNAs play critical roles in various cardiovascular diseases. Abnormal expression of *LncDACH1* leads to reduced calcium transient via promoting ubiquitin-related degradation of *SERCA2a* in heart failure [6]. What’s more, lncRNA *CPhar* cooperating with *DDX17* regulates *ATF7* by sequestering *C/EBPβ*, which is essential for exercise-induced physiological cardiac hypertrophy [7]. Nevertheless, limited knowledge exists in the lncRNA mechanisms related to AF.

Therefore, we attempted to explore lncRNA expression profiles in AF patients using RNA sequencing (RNA-seq). Subsequently, we validated differentially expressed lncRNA and established Gene Ontology as well as lncRNA-mRNA network to reveal the potential functions of lncRNAs in AF. Finally, we try to find novel biomarkers in AF.

## Materials and Methods

### Ethics statement

The study was performed in accordance with the Declaration of Helsinki. All study protocols and experiments were permitted by the Ethics Committee of the First Affiliated Hospital of the University of Science and Technology of China and prior informed consents were signed by participants.

### Study participants

In total, 97 patients were recruited in this study between March 2019 and August 2020, including 49 patients with AF (paroxysmal or persistent AF) and 48 patients without AF. Among these participants, 3 AF patients and 3 controls were used in RNA-seq and 91 patients (46 AF patients and 45 controls) were analyzed by quantitative Real-Time PCR (qRT-PCR). All AF patients were diagnosed by ECG or a Holter monitor according to the latest guidelines. The exclusion criteria of all patients were as follows: age < 18 years, valvular heart disease, heart failure with an ejection fraction < 40%, acute myocardial infarction or angina within 6 months, cancer, renal or liver failure, inflammatory diseases or hyperthyroidism.

### Blood sample collection

#### Erythrocyte lysis solution preparation and leucocyte extraction

Ammonium chloride (Sinopharm reagent, CHINA) 82g was added into distilled water (1000ml) to prepare the stock solution, and then the stock solution (100ml) was added into distilled water (900ml) to prepare the erythrocyte lysis solution stored at room temperature.

Peripheral venous whole blood samples (2ml) were placed into ethylenediaminetetraacetic acid (EDTA) anticoagulation tubes in the morning of the first day after admission. Blood samples were added with 13ml erythrocyte lysis solution, transferred into sterile dry centrifuge tubes and thoroughly mixed (155 rpm/min for 20min) to fully lyse the RBC within 2 hours. The mixtures were centrifuged (2200 rpm/min for 10minutes at 4℃) to separately obtain leucocyte precipitations which were then washed twice with Phosphate buffer saline (PBS).

#### Total RNA isolation

Total RNA of leukocytes was isolated using Trizol reagent (Invitrogen, Carlsbad, CA, USA) according to the manufacturer’s instructions. The quality and quantity of RNA were measured using the NanoDrop 1000 Spectrophotometer.

#### Reverse Transcription (RT), and Quantitative Real-Time PCR (QRT-PCR)

Reverse transcription was conducted by using HiScript II One Step RT-PCR Kit (Vazyme, CHINA) according to the manufacturer’s instructions. The cDNA was analyzed with real-time PCR using an SYBR Green qPCR kit (Vazyme, CHINA). Quantitative PCR was performed at 95^◦^C for 5 min following 40 cycles of 95^◦^C for 10 s and 60^◦^C for 30s in LightCyclerR 96 of Roche. The 2−ΔΔCt method was used to quantify the relative expression using GAPDH as the endogenous control. Primers used in this study are listed in Table S1.

### Transcriptome sequencing (RNA-Seq) analysis

A NanoDrop and Agilent 2100 bioanalyzer (Thermo Fisher Scientific, MA, USA) was used to measure the concentration and purity of total RNA. Ribosomal RNA (rRNA) was removed using target-specific oligos and RNase H reagents to deplete both cytoplasmic and mitochondrial ribosomal RNA from total RNA preparations. The RNA was used for custom DNBseq library preparation and sequenced on the DNBSEQ platform using PE100 mode. RNA isolation, library construction and RNA sequencing were performed by BGI-Shenzhen, China.

### Transcriptome sequencing data analysis

To analyze mRNAs and lncRNAs, the high-throughput sequencing tools, Hisat2 and feature counts, were used to map clean reads to Homo sapiens reference genome (hg19) and calculate the gene expression level which was normalized to z-score. To determine the differentially expressed mRNA and lncRNA, the “DEseq2” package in R software was used with the corresponding cutoff (*p* < 0.05, |log2(fold change)| > 1 for mRNA and lncRNA).

### Construction of lncRNA-mRNA Network

Based on significantly dysregulated lncRNAs and mRNAs, Pearson’s correlations among them were calculated according to the expression levels in six samples. A criterion of the correlations coefficient parameter R-squared more than 0.99 was used for the remaining RNAs to further construct the network.

### GO enrichment analysis

Gene ontology (GO) enrichment analysis was carried out to annotate the functions of differentially expressed genes with GO categories. GO enrichment analysis was implemented in R with the cluster profiler package. *P*-value < 0.05 was considered statistically significant.

### Alternative splicing analysis

rMATS [8] version 4.1.1 was conducted to compare alternatively spliced variants. Differential AS events including skipped exon (SE), mutually exclusive exon(MXE), alternative 5′ splice site (A5SS), alternative 3′ splice site (A3SS) and retained intron(RI) were identified between AF and control samples. Events with FDR < 0.1 and inclusion-level difference of >5% were considered differentially spliced across groups. The UpSet plot, generated by UpSetR [9], was conducted to quantitatively analyze the intersections between the five types of AS events in AF. Furthermore, to construct a splicing regulatory network, the Spearman correlation analysis was performed to evaluate the association between the splicing factors (SFs) and differentially spliced AS events. The list of 404 SFs was referred to a previous research [10]. *P* < 0.05 and correlation coefficient > 0.8 was the cutoff values. Finally, Cytoscape (version 3.8.0) was employed to build an SF-AS regulatory network.

### Statistical analysis

Continuous variables depending on normality were presented as the mean and standard deviation (SD), while categorical variables were expressed as counts and proportions. T-test and χ2 test were utilized to test the differences in continuous and categorical variables respectively. If continuous variables were not normally distributed, they were illustrated as median and interquartile ranges using Mann-Whitney non-parametric to compare. Receiver operating characteristic curves (ROC) and area under the ROC curve (AUC) were performed to assess the diagnostic value of lncRNA in AF patients. Logistic regression analysis was utilized to evaluate the risk or protective factor of AF. GraphPad Prism 5.0 and SPSS 21.0 were used for these analyses. *P*-value< 0.05 was significant statistically.

## Results

### LncRNA and mRNA expression profile

Three AF patients and 3 controls were enrolled for RNA-seq analysis, with 46 AF patients and 45 controls for validation of lncRNA expression. The baseline clinical characteristics of these groups are shown in Table 1.

**Table 1:** The clinical characteristics of the study patients and control subjects

	AF	Control	*P*-value
**Three pairs of patients for RNA-seq**			
N	3	3	-
Age, years	49.00±1.73	51.67±1.16	0.091
Male, n(%)	2(66.7%)	2(66.7%)	1
Smoking, n(%)	0(0)	0(0)	1
Hypertension, n(%)	0(0)	0(0)	1
Heart failure, n(%)	0(0)	0(0)	1
Diabetes, n(%)	0(0)	0(0)	1
CHD, n(%)	0(0)	0(0)	1
Stroke, n(%)	0(0)	0(0)	1
LAD(mm)	36.00±4.36	32.67±3.06	0.339
LVEDD(mm)	51.33±4.04	48.00±4.00	0.367
LVEF(%)	66.33±4.16	68.00±2.65	0.590
**Ninety-one patients for qRT-PCR validation**			
N	46	45	-
Age, years	62.83±10.44	54.27±13.37	0.001*
Male, n(%)	27(58.7%)	18(40%)	0.075
Smoking, n(%)	6(13%)	3(6.7%)	0.485
Hypertension, n(%)	26(56.5%)	15(33.3%)	0.026*
Diabetes, n(%)	10(21.7%)	3(6.7%)	0.040*
CHD, n(%)	4(8.7%)	2(4.4%)	0.677
Heart failure, n(%)	0(0)	0(0)	1
Stroke, n(%)	7(15.2%)	4(8.9%)	0.354
LAD(mm)	43.52±7.26	36.93±5.47	0.000*
LVEDD(mm)	50.54±4.63	49.93±4.67	0.533
LVEF(%)	65.30±6.57	66.51±6.63	0.385
AC009509.2	1.46 (0.71, 2.05)	0.44 (0.22, 1.46)	0.001*
LINC01781	0.59 (0.32, 0.90)	0.89 (0.45, 1.49)	0.011*

Values are n(%) for categorical variables and mean ± standard deviation for continuous variables

*Abbreviations: AF* Atrial fibrillation, *LAD* Left atrial diameter, *LVEDD* Left ventricular end diastolic diameter, *LVEF* Left ventricular ejection fraction, *CHD* Coronary heart disease

^*^P-values are from chi-square test for categorical variables and from t-test for continuous variables. * *p* < 0.05 is considered to be significant

**Fig. 1:**
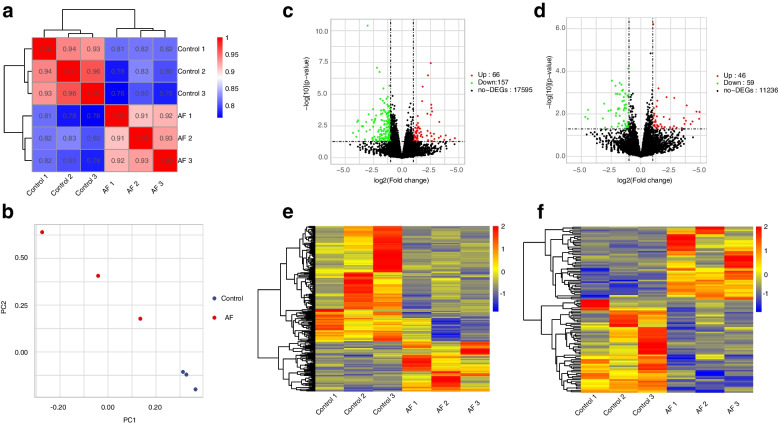
LncRNA and mRNA expression profiling (**a**) Sample correlation heatmap between control and AF group (**b**) Principal Component Analysis between control and AF group (**c**, **d**) The volcano plots of the significantly expressed mRNAs (**c**) and lncRNAs (**d**) (**e**, **f**) The hierarchical clustering heatmaps and scatter plots of differentially expressed (*p*< 0.05) mRNAs (**e**) and lncRNAs (**f**)

RNA-seq is a powerful tool to reveal the biological function of RNA. As shown in Fig. 1, replicates in AF and control groups correlate well and show a high correlation (>0.75) (Fig. 1a). Principal component analysis (PCA) is consistent with the heat map of sample correlation (Fig. 1b). We have identified differential expression of 223 mRNA genes and 105 lncRNA genes. Among them, 66 mRNAs were upregulated in the AF group, while 157 mRNAs were downregulated (Fig. 1c). There were 46 upregulated lncRNA and 59 downregulated lncRNAs in AF patients (Fig. 1d). Notably, the AF and control samples could be clearly differentiated from each other in the gene expression profiles (Fig. 1e, f).

### Validation of differently expressed lncRNAs

**Fig. 2:**
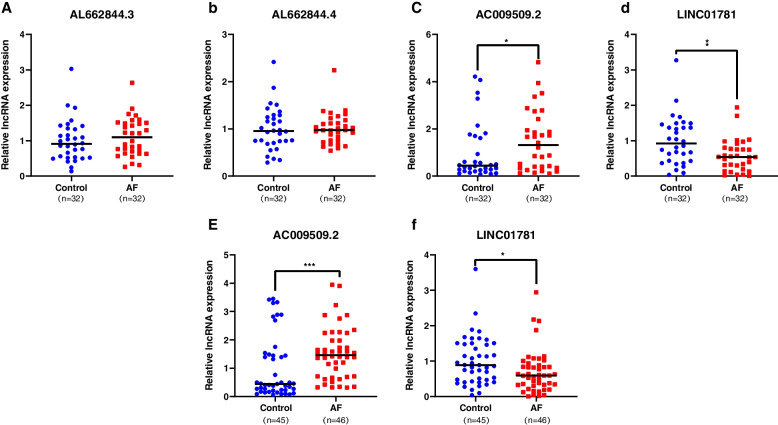
Different expression of lncRNA verified by qRT-PCR in 32 AF patients and 32 controls (**a**) The relative expression of AL662844.3 in AF and controls (**b**) The relative expression of AL662844.4 in AF and controls (**c**) The relative expression of AC009509.2 in AF and controls (**d**) The relative expression of LINC01781 in AF and controls * means *p*<0.05 Different expression of lncRNA verified by qRT-PCR in 46 AF patients and 45 controls (**e**) The relative expression of AC009509.2 in AF and controls (**f**) The relative expression of LINC01781 in AF and controls * means *p*<0.05

First, We found the mRNAs associated with atrial fibrillation on the website (www.genecards.org), and then intersected them with the mRNAs we obtained through RNA-Seq. Second, the same mRNAs were picked out and then we found the corresponding lncRNAs through the lncRNA-mRNA network. Finally, the same four lncRNAs (*LINC01781*, *AC009509.2*, *AL662844.3*, *AL662844.4*) were screened out. The method qRT-PCR was used to measure the expression levels of these four lncRNAs in 32 AF and 32 control groups (Fig. 2a-d) that was part of the 46 patients/45 controls. Two statistically different lncRNAs (*LINC01781 and AC009509.2*) were selected for continued qPCR testing. As demonstrated in Fig. 2e-f, *LINC01781* was decreased while *AC009509.2* was increased in the 46 AF participants when compared with the control group (*P* < 0.05). The other two lncRNAs were not statistically significant in 46 AF and 45 control participants (*P* > 0.05).

### GO enrichment analysis for the differentially expressed genes

**Fig. 3:**
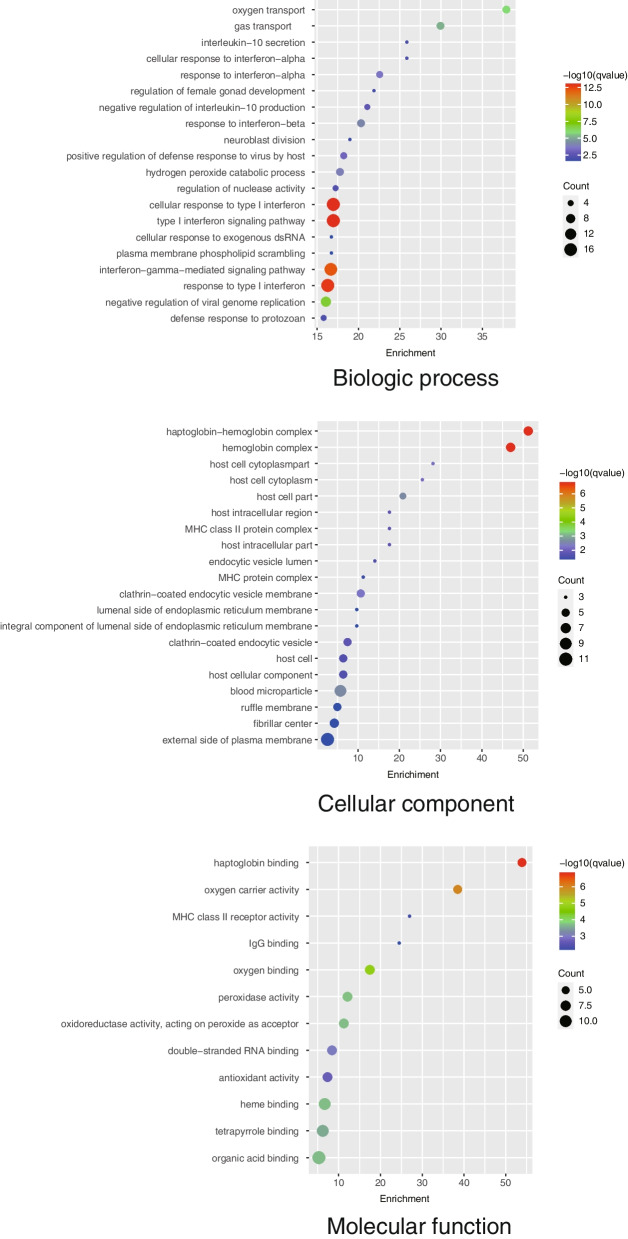
Significant GO enrichment terms related to differentially expressed genes

To investigate the possible roles of differentially expressed genes in AF, we performed GO enrichment analysis, which included biologic process, cellular component, and molecular function (Fig. 3). GO analysis showed that the biological process category involved mainly oxygen transport, gas transport, interleukin-10 secretion, and cellular response to interferon-alpha. Haptoglobin-hemoglobin complex, host cell cytoplasm, and MHC class II protein complex were the dominant cellular component terms. The molecular function category included mainly haptoglobin binding and oxygen carrier activity.

### Construction of lncRNA-mRNA network

**Fig. 4:**
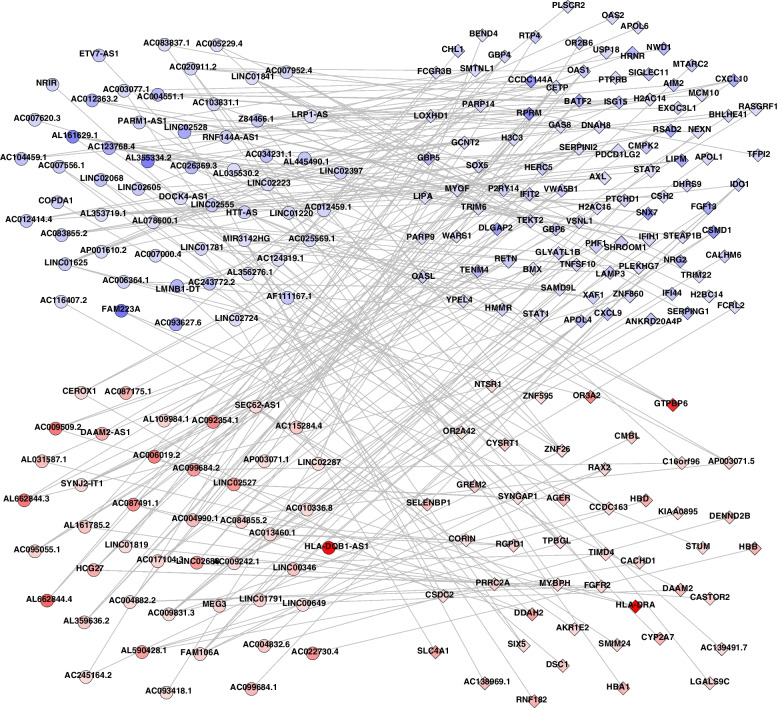
lncRNA-mRNA network of dysregulated gene in AF sample Circles represent lncRNAs and rhombi represent mRNAs, respectively. Red color indicates up-regulated RNAs, and blue color indicates down-regulated RNAs

To further explore the function of these dysregulated lncRNAs in the AF group, we attempted to construct a Pearson correlation matrix of the lncRNA-mRNA network between 223 differentially expressed mRNAs and 105 differentially expressed lncRNAs (Fig. 4). Associations were considered significant if the correlations coefficient parameter R-squared was more than 0.99.

### Validation of the key mRNAs and lncRNAs in the network with qRT-PCR

**Fig. 5:**
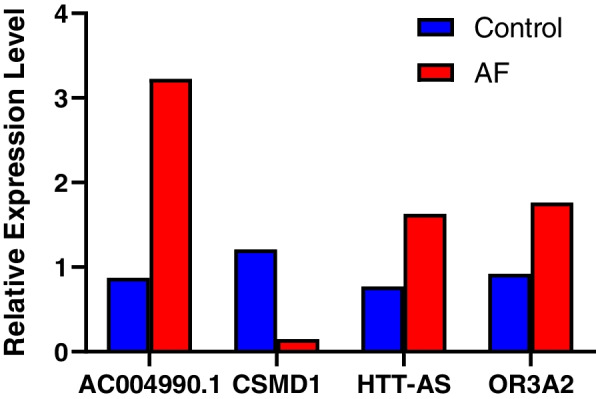
Differential Alternative Splicing Analysis (**a**) The UpSet intersection diagram shows five types differential AS events in AF (**b**) Correlation network between differentially expressed SF and PSI values of differential AS events generated by Cytoscape. Yellow ellipse was differentially expressed splicing factors. Red/blue ellipse were favorable/adverse AS events

The expression of four key mRNAs and lncRNAs (*CSMD1, OR3A2, HTT-AS* and *AC004990.1*) in the network were selected to validate in 11 AF patients and 11 controls by qRT-PCR. The expression profiles of these mRNAs and lncRNAs were consistent with the RNA-seq results except *HTT-AS* (Fig. 5).

### Differential alternative splicing analysis

**Fig. 6:**
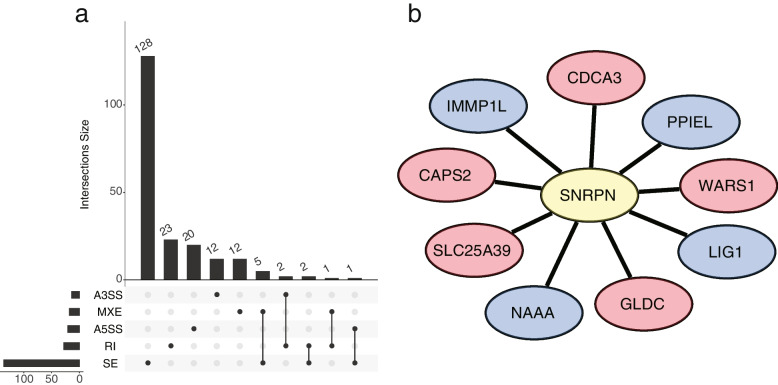
ROC curves for AF diagnosis (**a**)The value of LINC01781 for AF diagnosis in AF patients compared with the controls (**b**) The value of AC009509.2 for AF diagnosis in AF patients compared with the controls (**c**) The value of LAD for AF diagnosis in AF patients compared with the controls

Alternative splicing was considered as the mechanism that greatly contributed to the complexity of the genome and the variety of transcriptome [11, 12]. Thus, differential AS events were investigated by the rMATs paired model to determine the extent of alternative splicing changes in the AF patients. A total of 239 alternative splicing events for 206 genes were significantly different between AF and control groups (Fig. 6a), with SE being the most common, MXE and A5SS being the least. Besides, to reveal the underlying mechanism of AS regulation, we conducted a correlation network between the percent spliced in (PSI) values of AS events and the expression of SFs. However, only one SF called *SNRPN* was differentially expressed in our data. PSI values of five genes (*CAPS2*, *SLC25A39*, *WARS1*, *GLDC*, *CDCA3*) were positively correlated with the expression of SNRPN, while PSI values of four genes (*NAAA*, *IMMP1L*, *PPIEL*, *LIG1*) were negatively correlated (Fig. 6b).

### *AC009509.2* and LAD for AF patients in the aspect of diagnostic and predictive value

There was no significant difference in sex, smoking, heart failure, stroke, LVEDD or LVEF, while there were differences in age, hypertension, diabetes, LAD and the expressions of *LINC01781* and *AC009509.2* between two groups as shown in Table 1 and Fig. 2 (*P*<0.05).

**Fig. 7:**
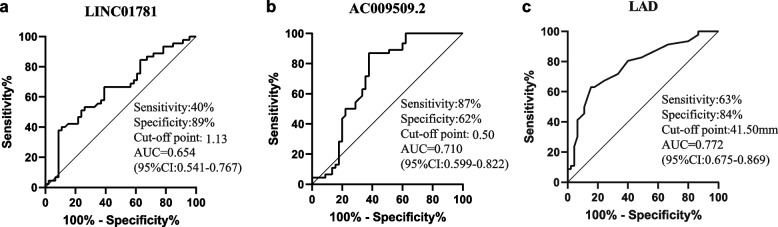
qRT-PCR validation of the key mRNAs and lncRNAs

The predictive value of *LINC01781*, *AC009509.2* and LAD was illustrated in Fig. 7. ROC analysis displayed that *AC009509.2* (AUC=0.710, sensitivity 87%, specificity 62%) and LAD (AUC=0.772, sensitivity 63%, specificity 84%) had a better diagnostic value for AF than *LINC01781* (AUC=0.654, sensitivity 40%, specificity 89%).

The univariate logistic regression analysis was demonstrated in Table 2, finding that age, hypertension, LAD and *AC009509.2* were risk factors for AF and *LINC01781* was a protective factor for AF. The multivariate logistic regression analysis found that *AC009509.2* and LAD were independent risk factors for AF as shown in Table 3.

**Table 2:** Univariate logistic regression analysis for AF vs controls

Characteristic	*β*	S·E	Wals	*P*-value	OR(95%CI)
Age	0.060	0.020	9.439	0.002*	1.062 (1.022-1.104)
Male	0.757	0.427	3.143	0.076	2.132 (0.923-4.921)
Smoking	0.742	0.741	1.003	0.317	2.100 (0.492-8.970)
Hypertension	0.956	0.434	4.845	0.028*	2.600 (1.110-6.088)
Diabetes	1.358	0.696	3.804	0.051	3.889 (0.993-15.225)
CHD	−0.717	0.893	0.644	0.422	0.488 (0.085-2.810)
Stroke	0.610	0.665	0.839	0.360	1.840 (0.499-6.780)
LAD(mm)	0.178	0.046	15.241	0.000*	1.195 (1.093-1.306)
LVEDD(mm)	0.029	0.046	0.397	0.528	1.029 (0.941-1.126)
LVEF(%)	−0.028	0.033	0.763	0.382	0.972 (0.912-1.036)
AC009509.2	0.532	0.224	5.675	0.017*	1.703 (1.099-2.639)
LINC01781	−0.788	0.373	4.458	0.035*	0.455 (0.219-0.945)

*Abbreviations: LAD* Left atrial diameter, *LVEDD* Left ventricular end diastolic diameter, *LVEF* Left ventricular ejection fraction, *CHD* Coronary heart disease

* *p* < 0.05 is considered to be significant

**Table 3:** Multivariate logistic regression analysis for AF vs controls

Characteristic	*β*	S·E	Wals	*P*-value	OR(95%CI)
Age	0.026	0.023	1.285	0.257	1.027 (0.981-1.074)
Hypertension	−0.128	0.562	0.052	0.819	0.880 (0.292-2.646)
Diabetes	−1.005	0.755	1.770	0.183	0.366 (0.083-1.609)
LAD(mm)	0.183	0.048	14.582	0.000*	1.201 (1.093-1.320)
AC009509.2	0.549	0.235	5.452	0.020*	1.732 (1.092-2.747)
LINC01781	−0.382	0.405	0.886	0.347	0.683 (0.308-1.511)

*Abbreviation: LAD* Left atrial diameter

* *p* < 0.05 is considered to be significant

## Discussion

AF is one of the most common clinical arrhythmias, leading to stroke and heart failure, and even death [13]. AF pathogenesis is associated with inflammation which leukocytes are the main cells involved in [14–17], but there are no effective biological markers. To date, an increasing number of researches showed that lncRNAs interfere with endogenous signaling pathways, playing a critical role in the development and progression of cardiac disorders [18].

Some scholars have found that the number of B cells is up-regulated, while the number of dendritic cells and T cell follicular assistance is down-regulated in atrial tissue of patients with atrial fibrillation, compared with that in the normal sinus rhythm group [19]. Meanwhile, given RNA expression profiles of blood cells have overlapping features with cardiomyocytes of hypertensive rats in the period of the preclinical and pathological stages of aldosterone/salt treatment [20] and blood leukocytes gene expression can be used to assess cardiovascular disease [21] we chose blood leukocytes to evaluate lncRNAs expression.

In our study, we identified lncRNA and mRNA expression profiles in leukocytes from three patients with AF and the other three patients without AF by RNA-seq. 223 mRNA genes and 105 lncRNA genes were significantly dysregulated with fold changes > 2. QPCR was used for further validation in another 46 AF samples and 45 control samples. In this study, blood leukocyte lncRNAs differentially expressed in AF groups were a higher level of *AC009509.2* and a lower level of* LINC01781.*

AF-related lncRNAs are a new area. Some previous studies have illustrated the different expression profiles of lncRNAs in AF [22, 23]. Mei et al. discovered different lncRNAs expressions in the right atrium tissues of rheumatic heart disease patients with AF compared with normal sinus rhythm. There were a total of 182 differentially expressed lncRNAs with 117 downregulated and 65 upregulated [22]. A recent study found that lncRNAs expression profiles in human lymphocytes from permanent AF differ from healthy controls [23]. Ke et al. analyzed public microarray datasets and constructed *LOC101928304/miR-490-3p/LRRC2* axis according to ceRNA theory in AF [24]. Besides, *LINC00844* has been shown to negatively regulated immune response in AF via affecting dendritic cells [19]. Above research results indicated that lncRNAs play an important role in the development and progression of AF.

Furthermore, we performed GO analysis to see functional changes in AF at the transcriptome level. Functionally, the dysregulated genes were significantly enriched in categories of oxygen transport, interleukin-10 secretion and cellular response to interferon-alpha. These categories might be associated with inflammation and metabolism. As indicated in many previous studies, the enhanced inflammatory response was associated with an increased risk of atrial fibrillation [25, 26]. Willeit K, et al. showed that soluble VCAM-1 involved in atrial remodeling [25]. What's more, NLRP3-inflammasome activation has been reported to promote AF pathophysiology [26]. The metabolic disease also plays a key role in AF. For example, metabolic dysregulation caused by mitochondrial dysfunction contributes to the pathogenesis of AF [27].

LncRNA-mRNA network was constructed based on the dysexpressed lncRNAs and mRNAs to reveal the potential roles of lncRNAs in AF. LncRNA *AC009831.3, AC123768.4, AL078600.1, AL161629.1, FAM106A, LINC01625, LINC01841, MEG3, SEC62-AS1* and *SYNJ2-IT1* showed the highest degree of connectivity, indicating their importance in the network. Among these lncRNAs, *MEG3* has been reported to suppress mTOR-mediated autophagy and inflammatory responses Cis-Diamminedichloroplatinum (II) -induced nephrotoxicity via negatively regulating miRNA-126 [28]. Nevertheless, further investigations are required to validate the regulatory functions of these lncRNAs.

Alternative splicing is a critical mechanism for the regulation of transcriptomes. Misregulation of alternative splicing is considered to be closely associated with a range of diseases. It is suggested that alternative splicing affects a broad range of cardiac genes during the pathological development of heart diseases. For example, *RBFOX1* is markedly decreased in failing human and mouse hearts. Lack of *RBFOX1* in the heart contributed to pressure overload-induced heart failure [29]. Our study identified 239 differential AS events for 206 genes between AF and the control group. Of note, a splicing factor called *SNRPN* was differentially expressed in our data and was significantly correlated with 9 different AS events. *SNRPN* is an imprinted gene that is selectively expressed in the brain and heart [30]. Previous studies identified that *SNRPN* is associated with several neurological disorders. It has been established that the paternal absence of *SNRPN* contributes to Prader-Willi syndrome [31, 32]. Besides, Zhao et al previously showed that aberrantly high methylation levels of *SNRPN* increase the risk of Congenital heart disease with extracardiac malformations via altering its gene expression [33]. However, the role of *SNRPN* in heart disease remains largely unknown.

*LINC01781* and *AC009509.2* were verified by qRT-PCR. The results revealed that *LINC01781* was downregulated while *AC009509.2* was upregulated in AF patients. *LINC01781* is an intergenic lncRNA located on chromosome 1, which involves cAMP signaling and interacts with neuroactive ligand-receptor according to the previous study [34]. *AC009509.2* is transcribed from chromosome 12, the function of which to date is not understood. Multiple logistic regression showed that only *AC009509.2* up-regulation was significant, considering that it may be related to the small sample size or that there may be an interaction between *LINC01781* and other factors. *AC009509.2* was screened out for this study, and there were very few studies on this gene at present, thus further studies on the mechanism of atrial fibrillation can be performed.

This study is limited by the small sample size and single-center design, so selection bias cannot be avoided. Larger research is needed to explore the lncRNAs function in AF. As blood leukocytes can partly reflect pathological conditions of cardiomyocytes, they are not equivalent. Further research is needed to base on atrial tissue.

## Conclusion

In this study, we searched for differential lncRNAs by the measurement of RNA sequencing in patients with atrial fibrillation, and then performed biological analysis. Finally clinical cases were used to verify the findings. Overall, *AC009509.2* was differentially expressed in AF patients, which may be new predictors and develop new therapy for AF.

### Supplementary Information

**Additional file1:** **Supplementary Table S1.** Primer sequence

## Data Availability

The datasets generated and/or analysed during the current study are available in the NCBI Sequence Read Archive (SRA) repository, [https://dataview.ncbi.nlm.nih.gov/object/PRJNA948158?reviewer=bclqgabu6ma5uh00dbn6sfruv8].

## Abbreviations

AF: Atrial fibrillation
lncRNAs: Long noncoding RNAs
RNA-seq: RNA sequencing
EDTA: Ethylenediaminetetraacetic acid
rRNA: Ribosomal RNA
GO: Gene ontology
AS: Alternative splicing
SE: Skipped exon
MXE: Mutually exclusive exon
A5SS: Alternative 5′ splice site
A3SS: Alternative 3′ splice site
RI: Retained intron
SFs: Splicing factors
SD: Standard deviation
ROC: Receiver operating characteristic curves
AUC: Area under the ROC curve
PCA: Principal component analysis
QRT-PCR: Quantitative real-time polymerase chain reaction
LAD: Left atrial diameter
LVEDD: Left ventricular end diastolic diameter
LVEF: Left ventricular ejection fraction
CHD: Coronary heart disease.
PBS: Phosphate buffer saline
PSI: Percent spliced in
MHC: Major Histocompatibility Complex
SRA: Sequence Read Archive
